# Studying the expression levels of LncRNA-MEG3 and miR-147b in the serum of psoriasis patients with and without dyslipidemia

**DOI:** 10.1038/s41598-026-52391-0

**Published:** 2026-05-19

**Authors:** Marwa Kamel, Rehab Elsayed Marzouk, Olfat G. Shaker, Mohammed Ali Gameil, Yasmine M. Amrousy, Esraa Farag, Hanan M. Abdallah, Amal Wagdy, Laila Mahdi

**Affiliations:** 1 Department of Medical Biochemistry and Molecular Biology, Faculty of Medicine, Capital University (Formerly Helwan University), Cairo, Egypt; 2 Department of Medical Biochemistry and Molecular Biology, Faculty of Medicine, Cairo University, Cairo, Egypt; 3 Department of Internal Medicine and Endocrinology, Faculty of Medicine, Mansoura University, Mansoura, Egypt; 4 Department of Clinical and Chemical Pathology, Faculty of Medicine, Capital University (Formerly Helwan University), Cairo, Egypt; 5 Department of Medical Microbiology and Immunology, Faculty of Medicine, Capital University (Formerly Helwan University), Cairo, Egypt; 6 Department of Physiology, Faculty of Medicine, Tanta University, Tanta, Egypt; 7 Department of Dermatology, Venerology and Andrology, Faculty of Medicine, Mansoura University, Mansoura, Egypt; 8 Department of Medical Biochemistry and Molecular Biology, Faculty of Medicine, Capital University (Formerly Helwan University), Cairo, Egypt

**Keywords:** Psoriasis, Dyslipidemia, Long non-coding, PASI, Biomarkers, Diseases, Genetics, Medical research

## Abstract

Psoriasis is a chronic systemic inflammatory skin disorder that is strongly associated with metabolic disorders, including obesity, insulin resistance, hypertension, and dyslipidemia. Dyslipidemia causes abnormalities in lipid profiles, such as elevated triglycerides or low density lipoprotein (LDL) levels. Measure maternally expressed gene 3 (MEG3) and microRNA-147b in serum of patients with and without dyslipidemia compared to healthy control. These measurements were compared with clinical characteristics, clinical parameters, and laboratory investigations. Serum expression levels of MEG3 and microRNA-147b, as non-coding RNA biomarkers, were measured across four equally subcategorized groups (patients with and without dyslipidemia (*N* = 66) and healthy controls with and without dyslipidemia (*N* = 66) using Polymerase Chain Reaction (PCR). Psoriatic patients exhibited a high significantly body mass index and elevated low-density lipoprotein levels compared with controls (*p* = 0.038,0.010) respectively, while fasting blood glucose was significantl at *p* = 0.001. Fasting blood glucose and lipid parameters showed low levels in patients without dyslipidemia and high levels of high-density lipoprotein than those with dyslipidemia. Expression Level of MEG3 was significantly downregulated, whereas microRNA-147b was markedly upregulated in psoriatic patients (*p* < 0.0001). MEG3 expression was negatively correlated with disease severity indices and lipid levels and inversely correlated with microRNA-147b, which also negatively correlated with disease duration. MEG3 and miR-147b, as non-coding RNAs, may show differential expression among psoriasis patient subgroups and could be associated with disease-related parameters, but their value as markers of disease severity or prognosis remains to be confirmed.

## Introduction

Psoriasis is a chronic inflammatory skin disease characterized by erythematous scaly plaques. It results from complex interactions between immune cells and keratinocytes, leading to excessive keratinocyte proliferation and dysregulated immune responses^1,2^.

Non-coding RNAs, including long non-coding RNAs (lncRNAs) and microRNAs (miRNAs), play important roles in psoriasis through regulation of inflammatory pathways and keratinocyte proliferation^3^. Long non-coding RNA maternally expressed gene 3 (MEG3) is a regulatory RNA involved in controlling cell proliferation, apoptosis, and inflammation, and has been reported to be downregulated in psoriasis, suggesting a potential anti-inflammatory role in disease pathogenesis. The long non-coding RNA MEG3 has been widely recognized as a tumor suppressor with regulatory roles in cell proliferation, apoptosis, and immune-related pathway^4,5^.

MicroRNA-147b (miR-147b) is involved in the regulation of inflammatory and immune responses and has been detected in circulation, with altered expression reported in inflammatory conditions. In psoriasis, its expression has been associated with disease-related parameters, indicating a potential role in reflecting systemic inflammatory status^6^.

Given that both MEG3 and miR-147b are implicated in inflammatory regulation and are detectable in serum, they were selected to explore their potential association with psoriasis in the context of metabolic disturbances, including dyslipidemia. The present study therefore aimed to evaluate their serum expression levels in psoriasis patients with and without dyslipidemia, compared to healthy controls, and to assess their association with clinicopathological parameters. Any relationship between these markers was examined at a correlational level without implying a direct mechanistic interaction.

## Subjects and methods

This is a comparative cross-sectional case-control study with a total of 132 individuals subdivided equally into four groups:

- Psoriasis patients with dyslipidemia (*N* = 33).
- Psoriasis patients without dyslipidemia (*N* = 33).
- Healthy control with dyslipidemia (*N* = 33).
- Healthy control without dyslipidemia (*N* = 33).

The study protocol was approved the Medical Research Ethics Committee, Institutional Review Board (IRB), Faculty of Medicine, Mansoura University, with approval code R.25.07.3264 on 25/08/2025. All methods were performed in accordance with the relevant guides and regulations and in accordance with the Declaration of Helsinki 1975). A written informed consent was obtained from all participants before the start of the study. Psoriasis participants fulfilling the eligible criteria were included in this study based on the sample-sized equation below^7^:

1 $$\:\mathrm{N}=\frac{{{\mathrm{Z}}_{1-\frac{\alpha}{2}}}^{2}\:\mathrm{p}(1-\mathrm{p})}{{\mathrm{d}}^{2}}\:=\:32.18$$

The accepted margin of error was set at 9.17% and a confidence level of 95%, the expected total sample size is 32.18, and we agreed on 33 individuals to be included in each group for the present study with threshold of significance 0.05.

Patients were recruited from the outpatient clinics and; or the inpatient wards of the Internal Medicine and Endocrinology Department and the Dermatology and Andrology Department, Mansoura University Hospitals.

All individuals included in the present study are adult. All patients were diagnosed with Plaque psoriasis. A minority (~ 15%) of patients with dyslipidemia demonstrated additional clinical variants, including flexural and pustular forms; however, all cases were included under the same diagnostic category for analysis.

Individuals with comorbidities such as Diabetes mellitus and Hypertension were not excluded; however, their potential confounding effects were evaluated and showed no significant impact on the studied parameters. The exclusion criteria included, patients under 18 years; any other skin disorder or autoimmune diseases. A full dermatological and clinical examination was done to all cases. Patients were not subjected to any drug history for ≥6 months. PASI score was assessed and calculated for all patients for detection the severity of psoriasis according to^8^.

### Real-time quantitative polymerase chain reaction (qPCR) for the expression of lncRNA-MEG3 and miR-147b

Total RNA including noncoding RNAs was extracted from serum using the miRNeasy Mini Kit with QIAzol Lysis Reagent (Qiagen, Valencia, CA, USA) according to the manufacturer’s protocol. RNA concentration and purity were assessed using a NanoDrop^®^ ND-1000 spectrophotometer (Thermo Fisher Scientific, Waltham, MA, USA). Extracted RNA samples were stored at − 20 °C until analysis.

Reverse transcription was performed using the miScript II RT Kit (Qiagen, Valencia, CA, USA) in a thermal cycler according to the manufacturer’s instructions.

Quantitative real-time PCR (qPCR) was performed using the miScript SYBR^®^ Green PCR Kit (Qiagen, Valencia, CA, USA) in a total reaction volume of 25 µL with specific primers for markers. Amplification was carried out using a real-time PCR system. GAPDH and U6 were used as internal controls for both MEG3 and miR-147b respectively. Fold change was calculated using the comparative threshold cycle (2^−ΔΔCt^) for relative quantification normalized to an endogenous controls^9,10^.

#### Statistical analysis of data

Statistical analysis was performed using SPSS software version 22.0 (IBM SPSS Inc., Chicago, IL, USA). Data were tested for normality using the Kolmogorov-Smirnov test. Continuous variables were expressed as mean ± standard deviation (SD) for normally distributed data or median with interquartile range (IQR) for non-normally distributed data. Comparisons between two independent groups were performed using the independent Student’s t-test for parametric data and the Mann–Whitney U test for non-parametric data. One-way ANOVA and Kruskal–Wallis tests were used for comparisons among more than two groups, as appropriate.

Categorical variables were analyzed using the Chi-square test. Correlations between variables were assessed using Pearson or Spearman correlation coefficients depending on data distribution. Multivariable logistic regression analysis was conducted to assess the association between biomarker expression levels and dyslipidemia status among psoriasis patients. Dyslipidemia was treated as a binary dependent variable (0 = absence, 1 = presence). Statistical significance was set at *p* ≤ 0.05.

## Results

In the present study 66 patients diagnosed with psoriasis covering 23 (34.8%) females and 43 (65.2%) males with a mean age of 44.79 ± 12.18 years; other 66 healthy controls of 22 (33.3%) and 44 (66.7%) females and males respectively with a mean age of 39.91 ± 10.88 were included. No significant difference was observed in age and sex ratio among the two groups (*p* > 0.05). For both groups, the body mass index value (BMI); comorbidity as well as laboratory tests were performed including Fasting Blood Sugar (FBS), Low-Density Lipoprotein (LDL), High-Density Lipoprotein (HDL), Triglycerides (TG) and Cholesterol (Table 1).

**Table 1:** Demographic, comorbidities and laboratory tests of the studied groups.

Variables	Psoriasis (*N* = 66)	Control (*N* = 66)	*P*-value
Demographic and descriptive data			
Age (years)	44.79 ± 12.18	39.91 ± 10.88	0.18
Gender (Female: Male)	23 (34.8%): 43 (65.2%)	22 (33.3%): 44 (66.7%)	> 0.05
Smoking (No: Yes)	38 (57.6%): 28 (42.4%)	54 (81.8%): 12 (18.2%)	> 0.05
Family History (No: Yes)	60 (90.9%): 6 (9.1%)	66 (100%): 0	> 0.05
Body mass index			
BMI value (kg/m^2^)	30.63 ± 5.16	28.85 ± 4.46	0.038*
Underweight	0 (0.0%)	2 (3.0%)	> 0.05
Healthy Weight	8 (12.1%)	8 (12.1%)	
Overweight	25 37.9%)	32 (48.5%)	
Obesity	33 (50.0%)	24 (36.4%)	
Waist circumference (cm)	103.52 ± 13.79	97.21 ± 11.94	0.17
Comorbidities			
DM (Negative:Positive)	48 (72.7%): 18 (27.3%)	56 (84.8%): 10 (15.2%)	> 0.05
HTN (Negative:Positive)	59 (89.4%): 7 (10.6%)	55 (83.3%): 11 (16.7%)	> 0.05
Laboratory test			
FBS (mg/dL)	100.12 ± 27.71	108.50 ± 53.39	0.001*
LDL_(mg/dL)	110.83 ± 43.82	89.35 ± 34.86	0.010*
HDL (mg/dL)	46.29 ± 16.40	45.41 ± 11.32	0.66
TG_(mg/dL)	158.94 ± 90.08	161.98 ± 73.21	0.60
Cholesterol (mg/dL)	192.98 ± 49.50	186.21 ± 127.62	0.07

Independent samples t-test is used for age and laboratory data. BMI: Body Mass Index; DM: Diabetes Melitus; HTN: Hypertension; FBS: Fasting Blood Sugar; LDL: Low-Density Lipoprotein; HDL: High-Density Lipoprotein; TG: Triglycerides.* Significant at *p*≤0.05.

Results revealed a significant difference between psoriatic patients and control group as regards to BMI (*p* = 0.038). Regarding laboratory investigations, FBS and LDL levels showed significant differences between patients group and healthy controls with *p* = 0.001 and 0.010 respectively (Table 1).

For both subgroup, a full clinical manifestations as well as laboratory investigations were measured, and recorded including skin Type; type of psoriasis; dermatological and features manifestations such as nail affection, pitting and subungual hyperkeratosis. Clinical Scores and Indices were also calculated for both groups (Table 2).

**Table 2:** Clinical, dermatological manifestations for psoriatic patients with and without dyslipidemia.

Variables	Psoriasis without dyslipidemia (*N* = 33)	Psoriasis with dyslipidemia (*N* = 33)	*P*-value
Skin type (I:II)	24 (72.7%):9(27.3%)	17(51.5%):16(48.5%)	0.076
Age of onset (months)	33.21 ± 13.64	37.52 ± 13.52	0.84
Disease duration (months)	60.0 (104.0)	72.0 (108.0)	0.10
BMI value (kg/m^2^)	28.99 ± 5.31	32.27 ± 4.50	0.42
Type_of_psoriasis			
Vulgaris	33 (100%)	23 (69.7%)	0.0001*
Vulgaris+Flexural	0	6 (18.2%)	
Vulgaris+Pustular	0	4 (12.1%)	
Dermatological features			
Nail affection (negative: positive)	26 (78.8%):7(21.2%)	16(48.5%):17(51.5%)	0.02*
Pitting (negative: positive)	28 (84.85%):5 (15.15%)	24 (72.7%):9(27.3%)	0.36
Subungual hyperkeratosis (negative: positive)	29 (87.88%):4 (12.12%)	24 (72.7%):9(27.3%)	0.4
Clinical scores and indices			
BSA_%	3.0 (13.0)	6.0 (13.0)	0.23
PASI_Score	2.2 (3.40)	4.0 (6.05)	0.017*
DLQI_Score	8.88 ± 4.92	10.12 ± 4.22	0.89
Laboratory investigation			
FBS (mg/dL)	of 85.39 ± 7.483	114.85 ± 32.50	**< 0.0001**
LDL_(mg/dL)	98.43 ± 37.472	123.22 ± 46.68	**0.0006**
HDL (mg/dL)	53.30 ± 19.470	39.27 ± 8.129	**0.034**
TG_(mg/dL)	137.06 ± 68.002	180.82 ± 104.278	**0.036**
Cholesterol (mg/dL)	176.45 ± 46.212	209.52 ± 47.728	0.369

Data shown as mean±SD for age of Onset, BMI value, DLQI_Score Independent student t-test is used. Data shown as median (IQR) for Disease Duration, BSA: Body_surface_area; PSAI: Psoriasis Area and Severity Index; Mann-Whitney U-Test is used for non-parametric data.* Significant at *p*≤0.05.

For the clinical features and sites affected, almost all patients had more than site affected including trunk, scalp, nail, upper and lower limbs, palms, soles, genitalia as well as flexures. The majority of the patients were affected in the upper and lower limbs almost 31 patients covering 47.0%, followed by the scalp 17(21.7%) of patients. Nails 10 (15.2%), trunck 9(13.6%), genitalia 5(7.6%), palms 4(6.0%) flexures 3(4.5%) and soles 2 patients (3.0%).

Table 2 shows full clinical, dermatological manifestations for psoriatic patients with and without dyslipidemia. All psoriatic patients without dyslipidemia were diagnosed with psoriasis vulgaris, whereas the patients with dyslipidemia exhibited multiple types of psoriasis such as flexural and pustular. A statistical significant difference between the distribution of patients as regards to the type of psoriasis was detected between both groups with *p* = 0.0001(Table 2).

It was noticed that in patients with dyslipidemia the patients were obese and overweighted covering 18(54.5%) and 15(45.5%) respectively. While for the group of psoriasis without dyslipidemia 8(24.2%) were classified as healthy weighted patients, 15(45.5%) obese and 10(30.3%) over weighted. A quite slightly statistically significant between the frequency of patients with respect to the BMI classification was detected at *p* = 0.05.

The laboratory data revealed that FBS levels tended to be significantly lower in patients without dyslipidemia at *p* < 0.0001 with mean and standard deviation of 85.39 ± 7.483 vs. 114.85 ± 32.50 for psoriatic patients without dyslipidemia versus psoriatic patients with dyslipidemia respectively. A significant difference was detected for the levels of LDL, TG for the two psoriatic patients groups with mean and standard deviations of 98.43 ± 37.472 vs. 123.22 ± 46.68 (*p* = 0.0006), 137.06 ± 68.002 vs. 180.82 ± 104.278 (*p* = 0.036). While cholesterol levels showed 176.45 ± 46.212 vs. 209.52 ± 47.728 with no significant difference between groups (*p* = 0.369). The level of HDL tended to be with high significant difference between patients without dyslipidemia and psoriatic patients with dyslipidemia with p-value of 0.034 with mean and standard deviation of 53.30 ± 19.470 than 39.27 ± 8.129 (Table 2).

**Fig. 1:**
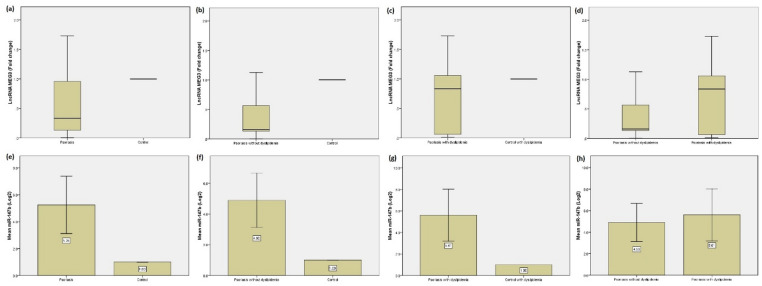
The expression levels of LncRNA MEG3 and miR-147b among the studied groups. (**a**) LncRNA MEG3 levels for psoriasis patients group and healthy control (**b**) LncRNA MEG3 levels for psoriasis patients without dyslipidemia and control (**c**) LncRNA MEG3 levels for psoriasis patients with dyslipidemia and control with dyslipidemia (**d**) LncRNA MEG3 levels for psoriasis patients with dyslipidemia versus without dyslipidemia (**e**) MiR-147b levels for psoriasis patients group and healthy control (**f**) MiR-147b levels for psoriasis patients without dyslipidemia and control (**g**) MiR-147b levels for psoriasis patients with dyslipidemia and control with dyslipidemia (**h**) MiR-147b levels for psoriasis patients with dyslipidemia versus without dyslipidemia.

The expression levels of LncRNA MEG3 and miR-147b were measured and compared according between the studied groups. For all psoriatic patients either with or without dyslipidemia, the levels of MEG3 tended to decrease in patients group than healthy controls with median and interquartile of 0.33 (0.84) with range of 0.002–1.73, whereas control individuals levels were 1.0 showing a high statistically significant difference between groups (*p* < 0.0001). The levels of miR-147b showed a high significant increase between patients with psoriasis and control group with mean and standard deviation of 5.25 ± 2.13 versus 1.0 (Fig. 1).

When investigating the subgroups, results showed that for both biomarkers the levels tended to elevate in patients with dyslipidemia showing significant differences when comparing psoriatic patients with and without dyslipidemia (Table 3; Fig. 1).

**Table 3:** The serum expression levels of MEG3 (FC) and miR-147b (Log2) biomarkers among the studied groups.

Biomarkers	Psoriasis without dyslipidemia (*N* = 33)	Psoriasis with dyslipidemia (*N* = 33)	Control without dyslipidemia (*N* = 33)	Control with dyslipidemia (*N* = 33)	*p*-value
LncRNA MEG3 Fold change	0.15 (0.49) 0.002–1.12	0.83 (0.99) 0.010–1.72	1.0	1.0	**0.031** ^**a**^ **< 0.0001** ^**bc**^ 0.113^de^
miR-147b Log2	4.90 ± 1.77	5.61 ± 2.42	1.0	1.0	**0.025** ^**a**^ **< 0.0001** ^**bcde**^

Data shown as median(IQR: Interquartile range) for non-parametric data by using Mann-Whitney U-Test. Data shown as mean and standard deviation for parametric data by Independent samples t-test. a between Psoriasis without dyslipidemia and Psoriasis with dyslipidemia. b between Psoriasis without dyslipidemia and Control without dyslipidemia. c between Psoriasis without dyslipidemia and Control with dyslipidemia. d between Psoriasis with dyslipidemia and Control without dyslipidemia. e between Psoriasis with dyslipidemia and Control with dyslipidemia. * Significant at *p*≤0.05.

Regarding comorbidities, diabetes mellitus and hypertension were present among both patients and controls, with no statistically significant difference between the two groups. DM was present in 18 (27.3%) of those without dyslipidemia and 10 (15.2%) of those with dyslipidemia, while hypertension was detected in 7 (10.6%) and 11 (16.7%), respectively, with no significant differences between these subgroups.

The expression levels of MEG3 and miR-147b showed no statistically significant association with diabetes mellitus (*p* = 0.06 and 0.21, respectively) or hypertension (*p* = 0.28 and 0.19, respectively).

Within patients with dyslipidemia, MEG3 expression showed a median (IQR) of 0.33 (0.04–1.04) in patients without diabetes mellitus and 0.92 (0.28–1.46) in those with diabetes mellitus. For miR-147b, patients without diabetes mellitus showed levels of 5.82 (4.56–6.44).

Regarding hypertension, MEG3 levels were 0.57 (0.04–1.12) in patients without hypertension and 0.97 (0.33–1.04) in those with hypertension. In contrast, miR-147b levels were 6.43 (2.72–7.76) and 4.56 (2.34–4.93) in patients without and with hypertension, respectively.

Patients were subcategorized according to the clinical scores and indices to allow for a more detailed assessment of disease severity as well as to quantify how much the skin is affected by psoriasis lesions. Regarding body surface area (BSA%), psoriasis patients were divided into two groups: mild (13 patients) and moderate-to-severe (20 patients). Among patients with dyslipidemia, the majority were classified as moderate-to-severe (24 patients) versus mild group comprising 9 patients. Analysis of PASI score further reinforced the pattern of disease severity among the study population. In psoriasis patients without dyslipidemia, the majority were classified as mild (28 patients), while only five were categorized as moderate-to-severe. In contrast, among patients with dyslipidemia, 25 were classified as mild and eight as moderate-to-severe (Table 4).

**Table 4:** Association of MEG3 (FC) and miR-147b (Log2) biomarkers levels in serum of psoriatic patients and patients with dyslipidemia as regards to clinical scores and indices.

Variables	Psoriasis patients (*N* = 33)		*p*-value
	Mild (*N* = 13)	Moderate/severe (*N* = 20)	
**Body surface area (BSA)**			
MEG3 (FC)	0.56 (0.68)	0.16 (0.12)	0.019*
miR_147b (Log2)	4.59 ± 1.93	5.09 ± 1.68	0.48

Variables	Psoriasis with dyslipidemia (*N* = 33)		*p*-value
	Mild (*N* = 9)	Moderate/severe (*N* = 24)	
**Body surface area (BSA)**			
MEG3 (FC)	0.33 (1.40)	0.15 (0.96)	0.048*
miR_147b (Log2)	2.86 ± 1.27	4.96 ± 2.18	0.015*

Variables	Psoriasis patients (*N* = 33)		*p*-value
	Mild (*N* = 28)	Moderate/severe (*N* = 5)	
**PASI_score**			
MEG3 (FC)	0.23 (0.54)	0.16(0.20)	0.19
miR_147b (Log2)	4.75 ± 1.87	5.69 ± 0.64	0.031*

Variables	Psoriasis with dyslipidemia (*N* = 33)		*p*-value
	Mild (*N* = 25)	Moderate/severe (*N* = 8)	
**PASI_score**			
MEG3 (FC)	0.33 (1.40)	0.15 (0.82)	0.05*
miR_147b (Log2)	4.04 ± 2.26	5.48 ± 1.49	0.033*

Variables	Psoriasis patients (*N* = 33)		*p*-value
	No/small/ moderate effect (*N* = 24)	Very large extremely large effect (*N* = 9)	
**Dermatology_life_quality_index (DLQI)**			
MEG3 (FC)	0.68 (0.80)	0.16(0.34)	0.001*
miR_147b (Log2)	4.63 ± 1.7	5.60 ± 1.80	0.94

Variables	Psoriasis with dyslipidemia (*N* = 33)		*p*-value
	No/small/ moderate effect (*N* = 20)	Very large extremely large effect (*N* = 13)	
**Dermatology_life_quality_index (DLQI)**			
MEG3 (FC)	0.33 (0.92)	0.05 (0.82)	0.016*
miR_147b (Log2)	3.42 ± 1.88	5.88 ± 1.80	0.001*

Independent samples t-test is used for age of Onset, BMI value, DLQI_Score. Mann-Whitney U-Test is used for non-parametric data. BMI: Body Mass Index; BSA: Body_surface_area; PSAI: Psoriasis Area and Severity Index; DLQI: Dermatology_Life_Quality_Index.* Significant at *p*≤0.05.

The impact of psoriasis on quality of life was measured by the Dermatology Life Quality Index (DLQI), revealed substantial heterogeneity between patient subgroups. Patients were classified into two main categories: those reporting no/small,/or moderate effect on their quality of life, and those experiencing a very large or extremely large effect. Among psoriasis patients, 24 reported minimal-to-moderate impact, while nine reported very large or extremely large effects. In the dyslipidemia group, 20 reported minimal-to-moderate impact, whereas 13 reported very large or extremely large effects. These findings suggest that comorbid dyslipidemia may exacerbate the psychosocial burden of psoriasis (Table 4).

**Fig. 2:**
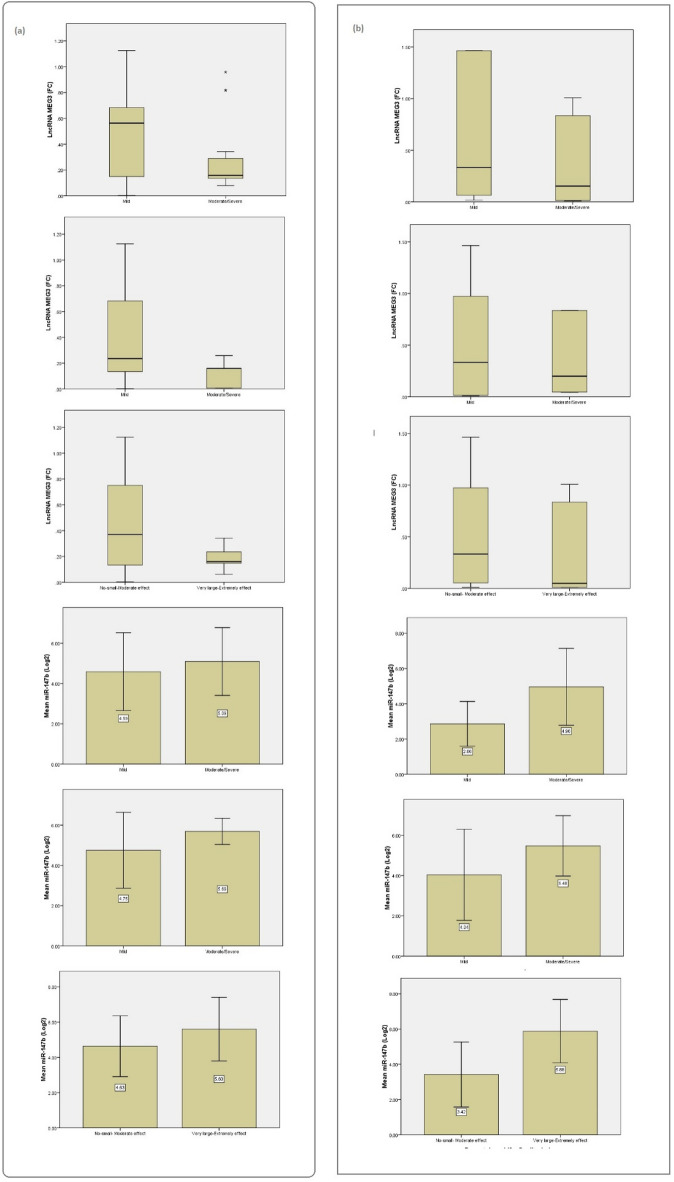
Association of MEG3 and miR_147b serum expression levels with clinical scores and indices (**a**) Box-plot diagrams for the levels of MEG3 as regards to BSA, PASI, DLQI subgroups, Bar-chart diagrams for and miR_147b levels as regards to BSA, PASI, DLQI subgroups for psoriasis patients without dyslipidemia. (**b**) Box-plot diagrams for the levels of MEG3 as regards to BSA, PASI, DLQI subgroups, Bar-chart diagrams for and miR-147b levels as regards to BSA, PASI, DLQI subgroups for psoriasis patients with dyslipidemia.

Biomarker analysis revealed distinct differences in serum MEG3 and miR-147b expression across severity groups. Regarding BSA classification, results revealed that in psoriasis patients without dyslipidemia the serum expression level of MEG3 showed a significant difference between mild and moderate-to-severe groups (*p* = 0.019). A slightly significant difference was also observed in the dyslipidemia cohort (*p* = 0.048). Similarly, miR-147B levels demonstrated a highly significant difference between severity groups (*p* = 0.015) in patients with dyslipidemia (Table 4; Fig. 2).

As for patients’ disease severity based on PASI classifications, results showed that MEG3 levels tended to decrease with the severity of the disease. While the level of miR-17b is elevating with the disease severity showing a statistical significant difference between groups with *p* = 0.031. For psoriasis patients with dyslipidemia, both MEG3 and miR-147B expression levels were significantly different between severity groups, with p-values of 0.05 and 0.033 respectively. These findings suggest that MEG3 and miR-147b may serve as potential biomarkers for distinguishing disease severity in psoriasis, both with and without comorbid dyslipidemia (Table 4; Fig. 2).

In addition when stratified by DLQI subgroups, MEG3 expression demonstrated a highly significant difference in psoriasis patients without dyslipidemia between those reporting minimal-to-moderate quality-of-life effects and those reporting very large or extremely large effects (*p* = 0.001). In psoriasis patients with dyslipidemia, MEG3 and miR-147b expression levels also differed significantly between the two DLQI subgroups, with p-values of 0.016 and 0.001 respectively. These results indicate that both serum biomarkers MEG3 and miR-147b expression levels not only correlate with clinical severity but also with the degree of quality-of-life impairment that may have potential roles as dual clinical and psychosocial biomarkers in psoriasis (Table 4; Fig. 2).

Results revealed that among patients without dyslipidemia, negative correlations were detected between the expression level of MEG3 and BSA percentage (*r*=-0.354,*p* = 0.043); PASI score (*r* = 0.361,*p* = 0.039) and with the DLQI score (*r*=-0.442,*p* = 0.010). Even regarding laboratory data, MEG3 showed a negative correlation with LDL level (*r*=-0.444,*p* = 0.010) and with serum cholesterol level (*r*=-0.553,*p* = 0.001). Additionally, a negative correlation was observed between miR-147b and MEG3 (*r*=-0.413,*p* = 0.017). There was also a negative correlation between miR-147b and disease duration (*r*=-0.423,*p* = 0.014).

Furthermore, the three scores were found to be correlated with various patient characteristics. For instance, The PASI score showed a positive correlation with BSA percentage (*r* = 0.587,*p* = 0.0001) and with the DLQI score (*r* = 0.611,*p* < 0.0001). Additionally, body mass index (BMI) exhibited a positive correlation with BSA percentage (*r* = 252, *p* = 0.044). PASI score showed a negative correlation with fasting blood sugar level (*r*=-0.35,*p* = 0.042) and a positive correlation with LDL level (*r* = 0.47,*p* = 0.006).

Among psoriatic patients with dyslipidemia, results revealed that serum level of MEG3 showed a negative correlation with waist circumference (*r*=-0.409,*p* = 0.018). For miR-147b, positive correlations were detected with BSA percentage (*r* = 0.508,*p* = 0.003), PASI score (*r* = 0.380,*p* = 0.029), and DLQI score (*r* = 0.504,*p* = 0.003). Regarding laboratory investigations, miR-147b was positively correlated with LDL level (*r* = 0.372,*p* = 0.033) and negatively correlated with HDL level (*r*=-0.410,*p* = 0.018).

Additional correlations were identified whereas a strong positive correlation between PSA score and BSA percentage (*r* = 0.912,*p* = 0.0001); a positive correlation between PSA percentage and DLQI score (*r* = 0.660,*p* = 0.001); and a positive correlation between PSA score and DLQI score (*r* = 0.532,*p* = 0.001). Furthermore, a negative correlation was observed between HDL level and DLQI score (*r*=-0.522,*p* = 0.002), as well as a positive correlation between TG level and DLQI score (*r* = 0.423, *p* = 0.014).

In addition, binary logistic regression analysis was performed to evaluate the association between MEG3 and miR-147b, considering as well confounding factors including BMI, diabetes mellitus, and hypertension for distinguishing psoriatic patients with and without dyslipidemia. The model was expressed as:

2 $$\:\mathrm{l}\mathrm{o}\mathrm{g}\mathrm{i}\mathrm{t}\left(\mathrm{p}\right)\:=\:\beta 0\:+\:\beta 1\:\mathrm{X}1\:+\:\beta 2\:\mathrm{X}2\:+\:\beta 3\:\mathrm{X}3\:+\:\beta 4\:\mathrm{X}4\:+\:\beta 5\:\mathrm{X}5$$

where p is the probability of being a psoriatic patients with dyslipidemi, X1-X5 denote MEG3, miR-147b, BMI, DM, HTN and β0-β5 are the estimated coefficients for each parameter as below:

3 $$\:\mathrm{l}\mathrm{o}\mathrm{g}\mathrm{i}\mathrm{t}\left(\mathrm{p}\right)\:=\:-2.10+2.41\left(\mathrm{M}\mathrm{E}\mathrm{G}3\right)+0.38(\mathrm{m}\mathrm{i}\mathrm{R}-147\mathrm{b})+0.05\left(\mathrm{B}\mathrm{M}\mathrm{I}\right)+0.20\left(\mathrm{D}\mathrm{M}\right)+0.30\left(\mathrm{H}\mathrm{T}\mathrm{N}\right)$$

The corresponding risk score was transformed into a predicted probability using:

4 $$\:\mathrm{p}\hspace{0.17em}=\hspace{0.17em}1\:/\:(1+\mathrm{e}^\wedge(-\mathrm{R}\mathrm{i}\mathrm{s}\mathrm{k}\:\mathrm{S}\mathrm{c}\mathrm{o}\mathrm{r}\mathrm{e}\left)\right)$$

After adding the confounding factors (BMI, diabetes mellitus, and hypertension), MEG3 and miR-147b remained significant independent predictors, whereas the other variables were not significantly associated with disease status. These findings suggest that the observed associations of the studied biomarkers are not solely attributable to metabolic or cardiovascular comorbidities (Table 5).

**Table 5:** Multivariate binary logistic regression analysis and model performance for the patients groups.

Marker/score	B	SE	Exp(B)	95% CI	*p*
MEG3 (FC)	2.41	0.72	11.18	1.30–4.20	0.001*
miR_147b (Log2)	0.38	0.15	1.46	1.14–3.05	0.016*
BMI	0.05	0.06	1.10	0.94–1.18	0.38
DM (0/1)	0.20	0.35	1.22	0.62–2.40	0.56
HTN (0/1)	0.30	0.40	1.35	0.61–2.95	0.45
Constant	-2.10	0.80	–	–	0.01*
Model indicator	-2 Log likelihood	Cox & Snell R Square		Nagelkerke R Square	
1	38.749	0.550		0.734	

Combined Risk Score = 0.751.

B: Coefficient, SE: Standard Error, Exp(B): Adjusted OR, 95% CI: confidence interval level of .for Exp(B) at 95%.

*Values are significant at *p* ≤ 0.05.

The multivariate model demonstrated good fit, with a -2 Log Likelihood of 38.74. The explanatory power of the model was moderate to strong based on Cox & Snell R² =0.550, and high based on Nagelkerke R²=0.73, indicating that approximately 73% of the variation in disease status could be explained by the included variables (Table 5).

A combined risk score incorporating both biomarkers was calculated, yielding a predicted probability cutoff of 0.751 (75.1%) with a statistically significant difference (*p* = 0.01). This combined model demonstrated improved diagnostic performance compared to each marker individually. Overall, these findings support the potential role of MEG3 and miR-147b as independent biomarkers associated with disease presence, with a strong overall predictive performance of the model (Table 5).

## Discussion

In the present study, patients with psoriasis showed a high significantl difference regarding BMI than healthy control. Although BMI was significantly elevated in psoriasis patients than control, the absolute difference between groups was relatively small. Moreover, BMI classification into categories did not differ significantly, indicating a comparable distribution of adiposity. In addition, waist circumference, a more reliable indicator of central obesity, showed no significant difference between groups. These findings suggest that the observed difference in BMI is unlikely to be clinically meaningful or to significantly influence the lipid profile and biomarker levels observed in this study.

FBS and LDL levels showed significant differences between patients group and healthy controls. When investigating the subgroups of psoriatic patients, FBS levels tended to be significantly lower in patients without dyslipidemia at *p* < 0.0001 than with dyslipidemia.

Levels of FBS showed a statistically significant difference between psoriasis patients and controls; however, the mean values were relatively close. The control group showed a relatively high standard deviation, suggesting variability in FBS levels among control cases, although still within reference ranges. In addition, subgroup analysis revealed higher FBS levels in patients with metabolic comorbidity compared with those without, with a statistically significant difference between the two subgroups. These findings suggest that metabolic alterations associated with chronic inflammatory and autoimmune conditions, as well as related metabolic disturbances, may influence glucose levels.

Even when further comparing psoriasis patients with and without dyslipidemia, findings showed that those with dyslipidemia demonstrated a significantly higher frequency of combined psoriasis subtypes (vulgaris with flexural or pustular involvement) compared with patients without dyslipidemia. Moreover, nail involvement was significantly more prevalent in the dyslipidemia group and patients with dyslipidemia also exhibited a significantly higher PASI score.

In agreement to our results, recent studies have shown that psoriasis is frequently associated with abnormal lipid profiles than healthy controls, including elevated triglycerides, elevated total cholesterol, increased low-density lipoprotein (LDL) cholesterol, and reduced high-density lipoprotein (HDL) cholesterol. These lipid abnormalities indicate a disturbed metabolic state that may influence skin cell function and closely linked with systemic inflammation and may influence disease expression and severity. Abnormal levels of these lipids can affect processes such as immune regulation and keratinocyte function, potentially contributing to more diverse clinical variability and the occurrence of non-vulgaris psoriasis subtypes among dyslipidemic patients. Altered lipid profiles have been reported in psoriasis and are associated with disease presence and severity. These associations suggest a possible link between metabolic dysregulation and psoriasis clinical variability, although the underlying biological mechanisms remain incompletely understood. Several studies have reported correlations between dyslipidemia and psoriasis phenotype and severity^11–14^.

A large cross-sectional study including more than 10,000 adult participants, of whom a subgroup had psoriasis, reported that patients with psoriasis had higher significantly obesity-related indices and unfavorable lipid profiles compared with non-psoriatic controls showing high significant as regards to BMI and LDL levels in psoriasis patients compared with the control group. This was in agreement to our findings where authors concluded that there was a high association between psoriasis and lipid accumulation and obesity indicators^15^.

In consistent with our finding, another research performed a systematic review and meta-analysis including more than 20,000 psoriasis patients across multiple studies to assess the association between psoriasis, dyslipidemia, and obesity. Authors demonstrated that psoriasis patients had significantly higher values of BMI even remarkable alteration in dyslipidemia parameters when comparing to patients without dyslipidemia relative to controls^13^.

In the present study, a significant difference was detected for the expression levels of MEG3 between psoriatic patients with and without dyslipidemia and between patients with dyslipidemia and healthy controls.

Conversely, miR-147b expression was significantly overexpressed in psoriatic patients compared with controls, with a further significant elevation observed in patients with dyslipidemia when compared to those without dyslipidemia.

The long non-coding RNA MEG3 normally helps control inflammation and keratinocyte growth in the skin. Both MEG3 and microRNA-147b may be functionally linked in psoriasis through a competing endogenous RNA (ceRNA) regulatory mechanism. MEG3 is generally considered an anti-inflammatory and anti-proliferative lncRNA that negatively regulates inflammatory signaling pathways, including NF-κB, and modulates cytokine production such as IL-6 and TNF-α^16^.

Downregulation of MEG3, which has been reported in psoriasis, may lead to loss of its miRNA-sponging capacity, resulting in increased activity of pro-inflammatory microRNAs such as miR-147b. miR-147b has been implicated in immune activation, inflammatory signaling, and cellular stress responses, and its upregulation may contribute to keratinocyte hyperproliferation and sustained cutaneous inflammation^4,17,18^. Together, reduced MEG3 expression and increased miR-147b activity could synergistically promote the chronic inflammatory characteristic of psoriasis.

In the present study, the expression levels of MEG3 and miR-147b were evaluated in psoriatic patients with and without dyslipidemia across patient’s severity. Among patients with moderate moderate/severe psoriasis, MEG expression showed a slight but statistically significant decrease compared with mild cases, irrespective of dyslipidemia status. In contrast, miR-147b expression was increased in psoriatic patients, with a high significant difference between those with dyslipidemia compared to those without dyslipidemia. This is consistent with previous reports highlighting the regulatory and tumor-suppressive functions of MEG3 across various pathological conditions^5^.

Similar trends were observed when patients were stratified according to the PASI score and the Dermatology Life Quality Index (DLQI). MEG expression showed a slight down-regulation in both patient groups, with down-regulation in MEG levels in the moderate/severe cases compared with the mild group. In contrast, miR-147b expression demonstrated an increasing trend across disease severity.

For the PASI score, miR-147b levels progressively increased from the mild group to the mild–severe group and further to the moderate–severe group. A comparable pattern was observed for the DLQI, where levels of miR-147b showed high significant difference between patients with very large to extremely large effects on quality of life compared with those with no/small/or moderate effects.

Notably, psoriatic patients with dyslipidemia exhibited statistically significant differences across all scoring systems when the categorized groups were compared collectively. Furthermore, when comparing psoriatic patients with and without dyslipidemia across all classifications based on PASI, PSA, and DLQI scores, patients with dyslipidemia showed a marked decrease in MEG expression accompanied by a slight increase in miR-147b expression relative to patients without dyslipidemia.

To the best of our knowledge, no studies specifically reported both MEG3 and miR-147b expression patterns in psoriatic patients stratified simultaneously by dyslipidemia status, BSA, PASI and DLQI. However, psoriasis has been reported as a systemic chronic inflammatory skin disorder with significant metabolic comorbidities, among which dyslipidemia is consistently more prevalent showing correlations between lipid abnormalities and with higher PASI scores in psoriasis patients, which supports our classification rationale in the present study^15,18,19^.

In psoriatic patients without dyslipidemia, MEG3 expression was negatively correlated with disease severity and disease-related impact (BSA percentage, PASI score, and DLQI score). MEG3 expression was also negatively correlated with lipid profile parameters (LDL and total serum cholesterol levels). In addition, the expression levels of MEG3 and miR-147b showed a negative correlation, whereby an increase in one was associated with a decrease in the other. Furthermore, miR-147b expression was negatively correlated with disease duration.

To the best of our knowledge, no studies to date have explicitly analyzed the statistical correlation between MEG3 expression and clinical severity or impact scores such as PASI, BSA, or DLQI in psoriasis patients with these clinical indices. However, several published articles have reported that MEG3 expression is significantly down-regulated in psoriasis tissue or lesions, and some have highlighted its potential mechanistic roles and diagnostic relevance in the disease. For example, MEG3 was found to be significantly downregulated in psoriatic skin samples and shown to regulate keratinocyte proliferation and apoptosis via interaction with miR‑21, indicating its functional involvement in psoriasis pathogenesis. Another recent study reported markedly reduced MEG3 expression in psoriatic lesions compared to healthy skin and identified MEG3 and miR‑21 as possible diagnostic markers for psoriasis vulgaris^20,21^.

Although no studies have directly examined the correlation between MEG3 expression and lipid parameters in psoriasis or other inflammatory skin diseases, evidence from other conditions shows that MEG3 can influence fat metabolism in cells. For example, in atherosclerosis, a chronic inflammatory cardiovascular disease, low MEG3 levels in foam cells are linked to increased fat accumulation, while in non-alcoholic fatty liver disease (NAFLD), a metabolic disorder, higher MEG3 levels reduce fat build-up in liver cells, helping prevent cell damage from excess fat. These findings suggest that MEG3 may help regulation of lipid levels, supporting our observation of a negative correlation between MEG3 and LDL or total cholesterol in psoriatic patients without dyslipidemia^22–24^.

In addition, our findings revealed that among psoriatic patients with dyslipidemia, MEG3 showed a negative correlation with waist circumference, while miR-147b showed positive correlations with disease severity and impact scores. Regarding lipid profile parameters, miR-147b was positively correlated with LDL and negatively correlated with HDL.

Until now, no study in dyslipidemia or in any other metabolic disorder has reported clinical correlations between MEG3 or miR-147b expression and lipoprotein parameters in relation to disease severity or clinical impact. Although MEG3 has been individually investigated in other metabolic conditions such as atherosclerosis^25^ and non-alcoholic fatty liver disease^26^, these studies mainly focused on its mechanistic role in lipid accumulation or cellular lipid handling rather than on correlations with circulating lipoproteins or clinical severity indices. Similarly, miR-147b has been reported in certain metabolic or metabolic-inflammatory disorders, including obesity-related inflammation^27^ and type 2 diabetes mellitus^28^, where its role was primarily linked to inflammatory pathways, without evaluating associations with lipoprotein parameters or disease severity. Importantly, none of these studies examined correlations between MEG3 or miR-147b and lipid profile parameters (such as LDL or HDL) in relation to clinical severity, highlighting the novelty of our findings in psoriatic patients with dyslipidemia.

In the present study, the levels of MEG3 and miR-147b showed a statistical association with the disease, as reflected by the significant difference in mean values as well as medians between groups. The observed association between MEG3 and miR-147b expression does not necessarily indicate a direct regulatory relationship. Both markers may be influenced by shared upstream regulators or may participate in parallel pathways related to inflammation and metabolic alterations in psoriasis and dyslipidemia. Further functional studies are needed to elucidate the underlying mechanisms and potential interactions.

The lack of significant association for diabetes and hypertension suggests that the observed biomarker effects are independent of these clinical factors Overall, while both markers can independently discriminate between psoriatic patients and controls, their combination does significantly enhance the diagnostic performance, with value of MEG3 and miR-147b in the combined model.

## Conclusion

The distinct expression patterns of lncRNA-MEG3 and miRNA-147b in psoriasis patients with and without dyslipidemia indicate that both biomarkers have potential value for discriminating between these patient groups. MEG3 levels tended to down-regulate in patients without dyslipidemia and partially restored in those with dyslipidemia, while miR-147b was elevated in all psoriasis patients and a slightly-higher in the dyslipidemic group. These findings suggest that the combined assessment of MEG3 and miR-147b could serve as a useful tool for identifying psoriasis patients with metabolic comorbidity.

## Data Availability

Yes. The data that support the findings of this study are not included in the manuscript for ethical reasons but are available upon reasonable request from the corresponding author, Olfat Shaker [email: olfat.shaker@kasralainy.edu.eg].
